# Design of Magnetic Hydrogels for Hyperthermia and Drug Delivery

**DOI:** 10.3390/polym13234259

**Published:** 2021-12-04

**Authors:** Sayan Ganguly, Shlomo Margel

**Affiliations:** Bar-Ilan Institute for Nanotechnology and Advanced Materials, Department of Chemistry, Bar-Ilan University, Ramat-Gan 52900, Israel

**Keywords:** biomedical applications, external stimuli, magnetic hydrogels, drug delivery, hyperthermia

## Abstract

Hydrogels are spatially organized hydrophilic polymeric systems that exhibit unique features in hydrated conditions. Among the hydrogel family, composite hydrogels are a special class that are defined as filler-containing systems with some tailor-made properties. The composite hydrogel family includes magnetic-nanoparticle-integrated hydrogels. Magnetic hydrogels (MHGs) show magneto-responsiveness, which is observed when they are placed in a magnetic field (static or oscillating). Because of their tunable porosity and internal morphology they can be used in several biomedical applications, especially diffusion-related smart devices. External stimuli may influence physical and chemical changes in these hydrogels, particularly in terms of volume and shape morphing. One of the most significant external stimuli for hydrogels is a magnetic field. This review embraces a brief overview of the fabrication of MHGs and two of their usages in the biomedical area: drug delivery and hyperthermia-based anti-cancer activity. As for the saturation magnetization imposed on composite MHGs, they are easily heated in the presence of an alternating magnetic field and the temperature increment is dependent on the magnetic nanoparticle concentration and exposure time. Herein, we also discuss the mode of different therapies based on non-contact hyperthermia heating.

## 1. Introduction

**Figure 1:**
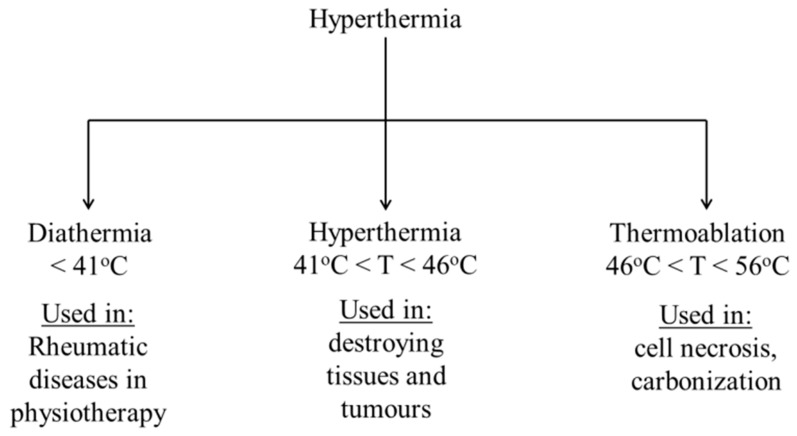
Classifications of hyperthermia and their uses.

The etymological definition of hyperthermia is the production of heat. Hyperthermia is a mode of cancer therapy that is implied to be a treatment carried out in presence of heat at the tumor region [1,2]. The apoptosis of tumor cells is affected by heat generation. Hyperthermia therapy is connected to magnetic nanoparticles, in which magnetic dipoles are stimulated in the presence of alternating magnetic fields, causing heat energy to be released [3,4]. Hyperthermia can be applied in various segments of medical therapies such as surgery, radiation therapy, gene therapy, chemotherapy, and cancer immunotherapy [5]. Hyperthermia is categorized into three categories based on how high the temperature rises as a result of it. The many kinds of hyperthermia are depicted in Figure 1. The low range of hyperthermia is defined as diathermia, where the temperature raised should not be beyond 41 °C [6]. The next stage of hyperthermia is moderate hyperthermia, which is the type of hyperthermia treatment used by doctors and physicians [7]. In moderate hyperthermia the temperature is in the range of 41–46 °C. When the temperature goes beyond 46°C it is then called thermal ablation [8]. In terms of applicability, cell necrosis and tissue necrosis occur at temperatures between 46 and 56 °C [9]. Low-temperature hyperthermia or diathermia is normally applied for rheumatic diseases, especially in physiotherapy [10]. In the moderate range of hyperthermia, i.e., in-between 41–46 °C, cellular proteins and DNA tend to be denatured, folded, agglomerated, and sometimes extensively cross-linked [11]. Due to these imbalances in the cellular proteins, gradual cell necrosis occurs [12]. Besides this, some other cellular effects are also seen as a result of moderate hyperthermia, such as the induction of apoptosis by signal transduction, multi-drug resistance, and heat shock protein (HSP) expression [13]. Hyperthermia has an impact that is not only reliant on the external magnetic field, but also on the exposure length, area of the targeted region, and cancer cell characteristics [14,15,16,17].

In terms of targeted hyperthermia therapy, this is also classified into three subsections, which are localized hyperthermia, regional hyperthermia, and whole-body hyperthermia [18]. These all depend on the exact position of the disease. Localized hyperthermia is a target-specific approach when the tumor is very small in size [19]. When the contaminated area is larger than the tumor, regional hyperthermia is used. This is utilized, in particular, for complete tissues or organs [20]. For individuals with cancer cells that have spread throughout the body, whole-body hyperthermia is employed. When compared to localized and normal hyperthermia, whole-body hyperthermia is more difficult [21]. In whole-body hyperthermia, there is a severe chance of the destruction of healthy tissues and organs.

Hyperthermia treatment traditionally takes place by administering external devices into tissues and transferring energy to irradiate them [22,23]. The transfers of energy for hyperthermia treatment are available in several works, such as electromagnetic waves, ultrasound, induction heating, radiofrequency, microwaves, infrared radiation, and magnetic nanoparticles being heated as thermo-seeds [24]. However, these strategies are constrained by their limits. Clinicians are proposing synergistic treatments that combine chemotherapy and radiation treatment [25]. This approach is superior because it does not affect healthy cells too much. There are some challenges experienced when performing hyperthermia treatment in a traditional way, such as the excessive heating of healthy tissues and severe discomfort due to blistering and burning, very low penetration of heat waves into the tissues with microwaves, lasers, and ultrasound, and sometimes a low dose of heating affects tumor cell apoptosis [26].

Magnetic-nanoparticle-based drug delivery is another area of research for the precise and controlled delivery of drug molecules to specific targets. The magnetic response feature of hydrogels is yielded by magnetic particles present in the hydrogels’ matrices [27]. Thus, magnetic nanoparticles’ inspiration is the most important step in the fabrication of MHGs. The magnetic behavior of MHGs and its associated performances are dependent on a few parameters, including the concentration of MNPs, ratio of polymer to MNPs, and size distribution of MNPs inside gel matrices [28]. There are several techniques adopted by materials scientists for preparing MHGs such as simple physical blending, in situ MNP formation, and grafting-onto methods [29]. According to the literature, natural polymers have limitations in regards to their ability to prepare MHGs because of the lack of abundance of active sites of the natural polymers [30]. Most of the natural-polymer-based MHGs are prepared either by blending or in situ approaches [31]. In the context of drug release behaviors of hydrogels, the primary mechanism is diffusion [32]. As hydrogels are porous networks, small molecules entrapped inside gel matrices come out into the environment. This drug release is facilitated by applying some external stimuli. Among them, a magnetic field is one of the applied stimuli. For MHGs, the externally applied field is classified into two categories; one is static and another one is dynamic or oscillatory [33]. In the case of an oscillatory field, an alternating current (AC) source is associated with the field, which fluctuates with frequency [34]. This is also called an alternating magnetic field (AMF). This release can be tuned by using two pathways adopted for the MHGs; one is by switching the external magnetic field and another one is altering the direction of the magnetic field [35]. When a field-driven arrangement is performed, the MNPs are aligned to form a barrier inside gel matrices [36]. Magnetic barriers impose a restriction to the capacity of small drug molecules to come out from the porous gel matrices, which signifies a lowering of the diffusion rate. Similarly, when the magnetic field is switched off the alignment is destroyed to some extent, followed by the release of drug molecules [37]. This is called magnetic pulsatile drug release behavior. Reports from various researchers also infer that the diffusion behavior has a direct relationship with the magnetic field strength.

## 2. Magnetic Nanoparticles (MNPs) in Hyperthermia

**Figure 2:**
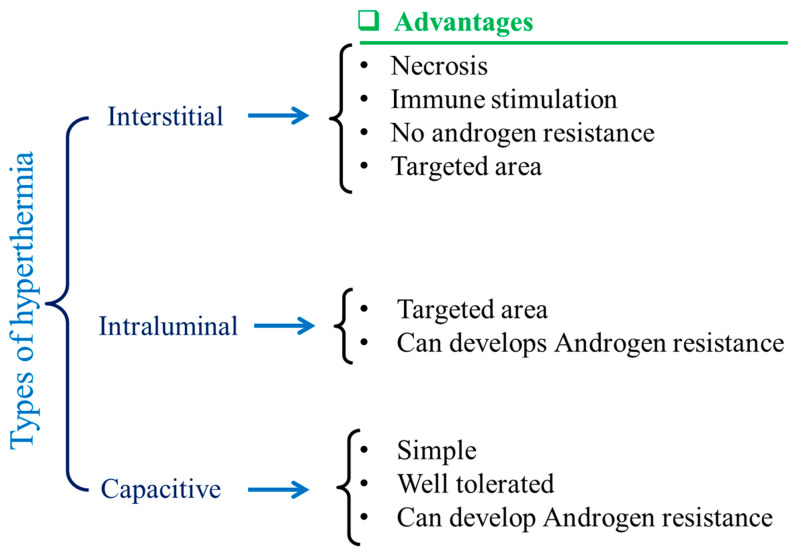
Schematic illustrations of different types of hyperthermia and their advantages.

Magnetic nanoparticles (MNPs) are dissipative centers of heat energy obtained from hyperthermia. This was first coined and applied in the year of 1957 [38]. Since then, MNP-based hyperthermia research has been on a fast track as MNP-based hyperthermia is quite superior compared to traditional hyperthermia in some aspects [39,40]. Various types of hyperthermia used by clinicians have been depicted in Figure 2. Traditionally, it can be divided into three areas as adopted by clinicians, such as intestinal [41], intraluminal [42], and capacitive [43]. The advantages are shown also in Figure 2. Tiny MNPs may easily pass through cell walls and, similarly to other nanoparticles, be heated in the presence of an external oscillating magnetic field [44,45,46]. This could cause more sophisticated and precise control of cell heating and necrosis. Sometimes MNPs can be functionalized by some target-specific molecules or surface engineering, which tend to attach to the cell walls [47]. This could make much better tissue-specific hyperthermia [48,49]. When MNPs are utilized, the oscillating magnetic field emits radiation that is only felt by the nanoparticles as opposed to the entire body, which is ideal for non-invasive therapy [50]. MNPs can easily permeate through the blood–brain barrier, which is desirable for glioblastoma treatment [51,52]. MNPs are also deliverable with drug molecules [53]. This feature could make MNPs dual-model nanoparticles for serving better therapeutic assays. MNPs are also superior for better dispersion in a target site compared to any bulk implantation. This could maintain the homogeneity of the heating during hyperthermia. Moreover, MNP heating is also effective for further anti-tumoral immunity [54]. Improved saturation magnetization of MNPs can also be achieved by synthesis optimization and layer-by-layer growth mechanisms. These characteristics may make them multimodal and therapy-focused nanoparticles [55].

The most common and widely used MNPs are magnetic (Fe_3_O_4_) [56,57] and maghemite (γ-Fe_2_O_3_) [58,59]. The most promising characteristic of MNPs is their non-cytotoxicity [60]. Maghmeite is the oxidized product of magnetite at high temperatures (~300 °C) [61]. However, magnetites (MNPs) are more common than maghemite MNPs due to the simplicity of their manufacture and purifying techniques, despite maghemite’s superior thermodynamic stability. As a result, magnetite-based hyperthermia was reported in the majority of MNP-based studies [62]. If the MNPs’ size lies within a few nanometers, such as 1–5 nm, cell permeation occurs easily [63]. Magnetic behavior is also dependent on the size and shape of MNPs [64]. For bulk, magnetic materials’ multi-domain presence is a common thing, but when the size of the material becomes lower the multi-domain particles become single-domain [56,65]. By this approach, multi-domain materials turn from ferromagnets into superparamagnets [66].

The hyperthermia mechanism can be classified into two broad segments; one is hysteresis loss and another one is Néel relaxation loss [67,68]. There is one aspect that these two systems have in common: they are not reliant on the optimal particle size. In terms of hysteresis loss behavior, multi-domain ferromagnets are inferior to single-domain ferromagnets. When compared to multi-domain ferromagnets, single-domain ferromagnets emit a substantial amount of heat. Hysteresis loss is not present in superparamagnetic nanoparticles. Superparamagnetic nanoparticles generate heat in the presence of alternating magnetic fields because of the relaxation loss phenomenon, especially Néel relaxation loss.

## 3. Hydrogels

Hydrogels are a special class of three-dimensional polymeric material composed of hydrophilic polymer chains [69]. Hydrogels are insoluble to any solvent, but their volume changes after water uptake [70]. Hydrogels are also affected by external stimulants such as pH, salt, an electric field, a magnetic field, and mechanical stress [71,72,73]. Hydrogels can be categorized into two classifications: chemical hydrogels and physical hydrogels. Chemical hydrogels are chemically cross-linked hydrophilic polymer chains which are intermingled in a polymer matrix [74]. Sometimes these are also called covalent cross-linked hydrogels [73,75,76]. Physical hydrogels are non-covalent hydrogels where different types of molecular forces of attraction form an insoluble gel mass [77]. The molecular forces which play a major role in gel formation are polar–polar interaction, hydrogen bonding interaction, hydrophobic association, and mechanical interlocking or entanglement [78]. Chemical hydrogels are always thermally stable compared to physical hydrogels according to the stability of physical and chemical hydrogel systems. Physical hydrogels can sometimes be reversible when exposed to high temperatures. Physical hydrogels are referred to as reversible hydrogels; chemical hydrogels are fully irreversible.

**Figure 3:**
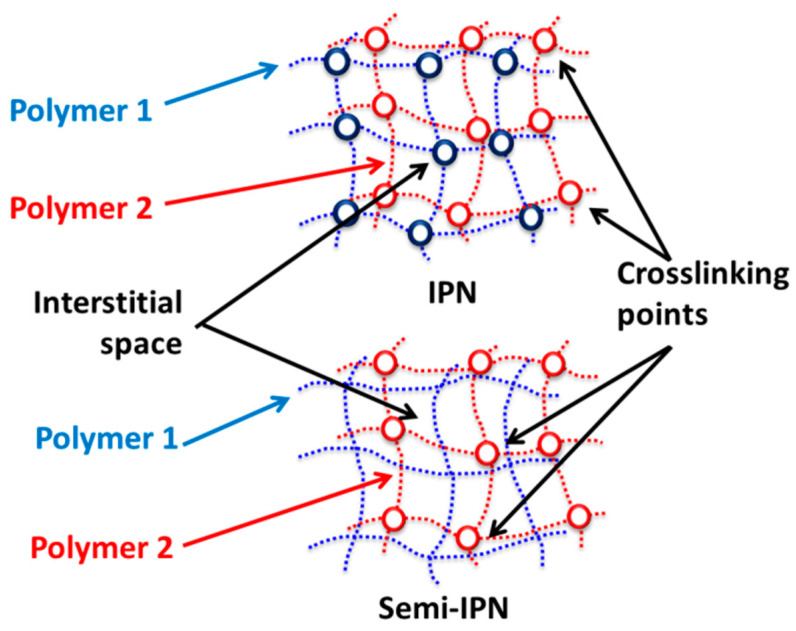
Schematic of IPN and semi-IPN systems. IPNs show cross-linking of both the polymer phases whereas the semi-IPN shows only one phase cross-linking.

Hydrogels containing at least two polymer phases when both the phases are cross-linked are called interpenetrating polymer networks (IPNs) (Figure 3). Similarly, when at least one polymer face is cross-linked and the other phase remains non-cross-linked the system is called a semi-interpenetrating polymer network (semi-IPN) [79]. When compared to semi-IPN systems, IPNs are always superior in terms of mechanical strength [80]. Gel matrices are very vulnerable to water, drug molecules, and a variety of hydrophilic small molecules due to the presence of hydrophilic polymer chains [81]. Hydrogels are composed of elastic as well as viscous components [82,83]. Any hydrogel’s viscous and elastic properties may be fine-tuned by altering the precursors, reaction parameters, and amount of water absorbed. Because of their porosity, regulated diffusion behavior, and great biocompatibility, hydrogels are used in a variety of medicinal applications [84]. Various types of implantations are already established by hydrogel-based scaffolds. These types of hydrogels are prepared by using either divinylic cross-linkers or physical entanglements. For divinylic cross-linking systems, IPNs possess high mechanical properties as well as high thermal stability [85,86]. In general, the cross-linkers utilized here are phase-selective. Divylic cross-linkers are commonly utilized for free-radical-triggered monomers. Ionic cross-linking has been done on polysaccharides such as alginate and carrageenans. Borax and glutaraldehyde are the most popular cross-linkers for polyvinyl alcohol (PVA) [87]. Chitosan-based biopolymers are cross-linked by NaOH [88] and genipin [89]. For NaOH-induced cross-linking of chitosan physical entanglement occurs, whereas for genipin the cross-linking is based on chemical routes. Because of their porosity, hydrogels are efficient platform for delivering cells and drugs in three-dimensional systems. However, hydrogels also suffer from several limitations, such as fast responsiveness and mechanical properties [90]. In the biomedical area, hydrogels play a crucial role in drug delivery and other therapeutics.

## 4. Fabrication of Magnetic Hydrogels

MHGs are a special class of hydrogel that contain at least one magnetic component in their composition. Generally, MNPs are dispersed in a polymer gel matrix to form MHGs. These hydrogels are special because they are prone to show fluctuations in their physical properties in the presence of an externally applied magnetic field. The magnetic behavior of MNPs employed in polymer gel matrices varies depending on their size. Scientists have used a variety of ways to create MHGs, including mixing, an in situ approach, grafting onto, and gelation in the presence of a magnetic field.

### 4.1. Fabrication of Magnetic Hydrogels by Blending

**Figure 4:**
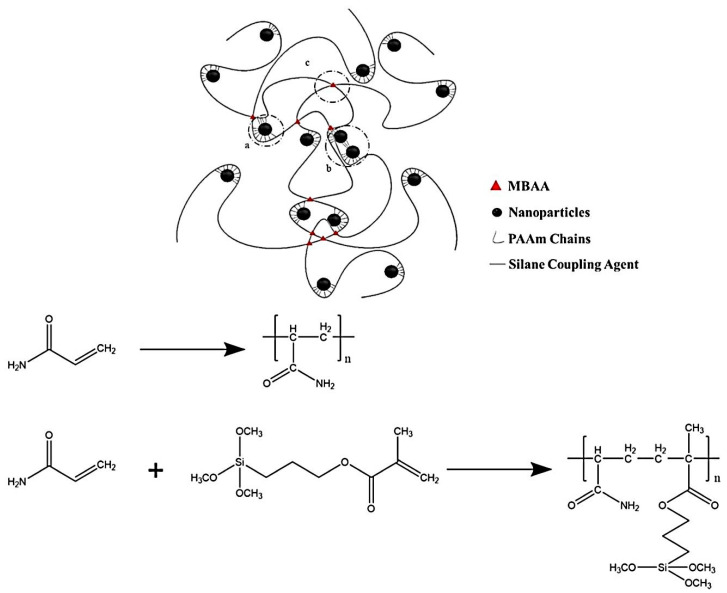
Schematic representation of MNP-blended hydrogel for soft robotics [96] © 2021 American Chemical Society.

The most common and easy method to fabricate magnetic composite hydrogels is blending. Fe_3_O_4_ is the most commonly used MNP blended with a polymer system to prepare composite hydrogels. This approach involves two steps: first, MNPs are produced and kept in an aqueous oil phase to prevent oxidation. The MNPs are then combined with hydrogel precursor materials before the polymer matrix is cross-linked. Tong et al. used a simple mixing approach to create a magnetite-loaded thermoresponsive hydrogel [91]. Such a hydrogel was tested against an externally applied magnetic field. In another work both magnetite and maghemite were prepared and loaded into a dextran hydrogel to produce a magneto-responsive composite hydrogel system via a photopolymerization approach [92]. Alginate-based magnetic beads were reported where maghemite was introduced to obtain a magnetic-field-responsive hydrogel [93]. The mix technique is a low-energy, quick-to-develop composite hydrogel. This approach may also be used to introduce MNPs with varied particle size distributions into hydrogel matrices. MHG beads were prepared using Fe_3_O_4_ MNPs for adsorption applications. Magnetite-nanoparticle-loaded beads were surface functionalized by gallic acid for the efficient removal of Cr(+6) [94]. In another work, methacrylate-functionalized hydrogel was reported where magnetite MNPs were mixed and utilized for hyperthermia treatment [95]. A high amount of MNPs (up to 60%) was also used for preparing tough MHGs [96]. Here, the MNPs were Fe_3_O_4_ and functionalized with 3-(trimethoxysilyl)propyl methacrylate to achieve crosslinking in the polyacrylamide phase. These hydrogels were used as soft robots when coated with a PDMS rubber support. The synthesis mechanism is shown in Figure 4.

### 4.2. Fabrication of Magnetic Hydrogels by the In Situ Method

The synthesis of in situ MNP-based composite hydrogels is more precise in terms of the particle size and architecture of the hydrogel [97]. In this procedure, MNPs are prepared inside the gel matrices; this process is carried out during the gelation. MNPs’ precursor metal ions are initially dispersed into the hydrogel precursor materials, especially the monomers [98]. The gelation process then produces an insoluble solid gel mass. The gel is then alkali-treated to obtain MNPs inside the hydrogel matrix [99]. Otherwise the in situ method was also carried out by adopting an ‘uptake and arrested’ strategy. In this method, a gel was immerged into MNPs’ precursor metal ions solution until equilibrium was reached [100]. Then, the metal-ion-entrapped swelled hydrogel was transferred into an alkali bath to grow MNPs inside the hydrogel matrix [101]. This causes an efficient distribution of MNPs inside the gel that could enhance homogenous saturated magnetization of the magnetic composite hydrogel.

Magnetite was prepared by the in situ method according to the following chemical reaction [102]:

Fe^2+^ + 2Fe^3+^ + 8OH^−^ → Fe_3_O_4_ + 4H_2_O (1)

It is apparent that the stoichiometry for the Fe^2+^/Fe^3+^ molar ratio should be kept at 1:2 when producing magnetite using this approach. Both the concentration of iron ions and the alkali utilized increase the yield of the product (MNPs) [103]. Nagireddy et al. reported composite MHGs obtained via an in situ coprecipitation method where the hydrogel matrix was gum-acacia-grafted polyacrylamide [104,105]. Semi-interpenetrating hydrogel (semi-IPN) was also created, in which Fe_3_O_4_ MNPs were deposited in situ in a poly(N-isopropyl acrylamide) matrix [106]. The MNPs contain several hydrophilic fictional groups that could enhance the physical cross-linking of the hydrogel [107]. Such additional cross-linking because of the MNPs affect the porosity of the composite hydrogel [108,109].

**Figure 5:**
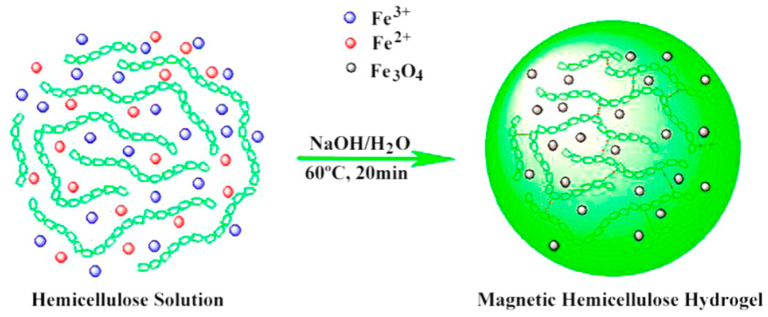
Synthesis of an in situ MNP-based magnetic hydrogel [111] © 2021 American Chemical Society.

Cellulose is a naturally abundant biopolymer [110]. A group of researchers reported on a hemicellulose-based in situ MNP hydrogel in which the MHG beads displayed outstanding magnetic-field-regulated drug release behavior [111]. Figure 5 shows the in situ synthesis of MNPs in a hemicellulose matrix.

### 4.3. Fabrication of Magnetic Hydrogel by the Grafting-Onto Method

**Figure 6:**
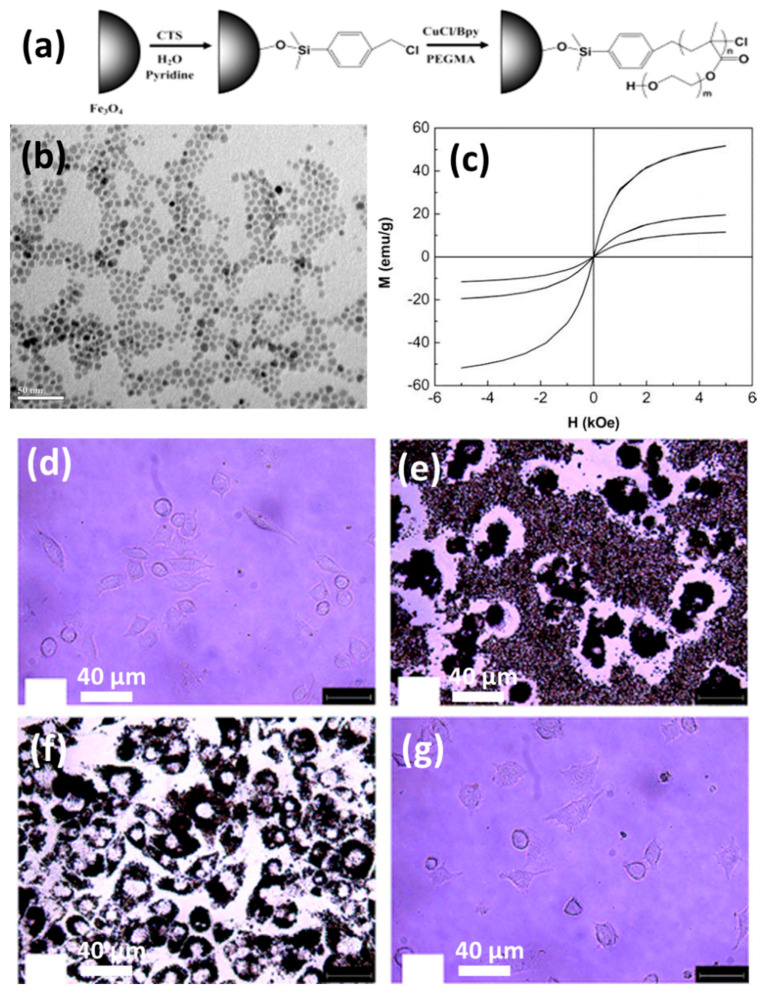
(**a**) Reaction scheme of the PEGylation of MNPs. (**b**) TEM image of MNPs. (**c**) Saturation magnetization plot for pristine and surface-decorated MNPs. (**d**) Monocyte/macrophage-like cells (RAW 264.7) in a control experiment. Cells after culturing in a medium containing pristine magnetic nanoparticles for (**e**) 1 day and (**f**) 4 days. (**g**) Modified MNPs in cultured media [112] © 2021 American Chemical Society.

The two approaches previously presented have one thing in common: they are both non-covalently arrested inside gel matrices. This procedure of grafting on is the polar opposite of them. MNPs are chemically changed in this experiment to increase their compatibility with the polymer system. The surface of MNPs is chemically bonded to the hydrogel polymer chains in the grafting-onto technique. In terms of stability, these MNPs outperform the physical or basic blending methods. In this case, the MNPs have been altered so that the small nanoparticles themselves operate as a cross-linker in the hydrogel system. Jahanban-Esfahlan et al., prepared functionalized magnetic nanoparticles where the surface ligands used were amino silanes [98]. The functionalized magnetic nanoparticles were then incorporated into a hydrogel matrix. At room temperature the hydrogel was pH-sensitive and displayed a regulated release of chemotherapeutic medicines. They also exploited medication release synergy with heat to improve accuracy and efficacy. Polymer-grafted magnetic nanoparticles are also drawing attention because of their compatibility and better tumor-area-targeting behavior. Polyethylene glycol is a typical surface-decorating polymer that has been used by a number of materials scientists. Hu et al. developed PEGylated magnetic nanoparticles that displayed 19 emu/g saturation magnetism [112]. In this work they used copper-mediated ATRP polymerization to obtain poly(ethylene glycol)-methacrylate-grafted magnetic nanoparticles. The surface polymer was used for easy anchoring of protein molecules. The synthesis is graphically illustrated in Figure 6a. The morphology and saturation magnetization plots are also given in Figure 6b,c, respectively. The cells with MNPs are also shown in Figure 6d–g. These images show how the MNPs performed against cells. It is clear and evident that there were no cellular distortions after 4 days. The MNP uptake also occurred without any cell death. Atrei et al. developed CoFe_2_O_4_-based MHGs, where they chemically modified the MNPs with carboxymethyl cellulose (CMC) [113]. Similarly, Schmidt et al. prepared a polyacrylamide-grafted CoFe_2_O_4_-MNP-based hydrogel by the covalent coupling method [114]. These works suggest that if the MNPs are surface functionalized they are very compatible with hydrogel matrices.

The properties of MHGs, excluding their magnetic behavior, are dependent on the magnetic nanoparticles [115]. Mechanical strength, reinforcement, and cross-linking are provided by the magnetic nanoparticles. In nature, hydrogels made from natural polymers are biodegradable. Magneto-sensitive hydrogel systems are already made from a variety of natural polymers. Table 1 shows some composite MHGs that have been used in biological applications. If the magnetic nanoparticles do not mix well with the hydrogel matrix they have poor mechanical characteristics, limiting their application as tissue-mimicking biomaterials. In comparison with natural polymers, synthetic polymers have higher mechanical strength. The volume fraction of magnetic nanoparticles inside hydrogel matrices has some significant role in controlling the saturation magnetization, and this can be described as for the following equation [116]:

(2) $$M=\varphi_{m}M_{s}\left(coth\xi -\frac{1}{\xi }\right)$$

Here, *φ_m_* is the volume fraction of the magnetic nanoparticles in the hydrogel matrix. *M_s_* is the saturation magnetization. *ξ* corresponds to mH/k_B_T, with m, H, k_B_, and T representing the magnetic moment of the MNPs, the external magnetic field, the Boltzmann constant, and the temperature, respectively.

## 5. Hyperthermia-Based Cancer Treatment

**Figure 7:**
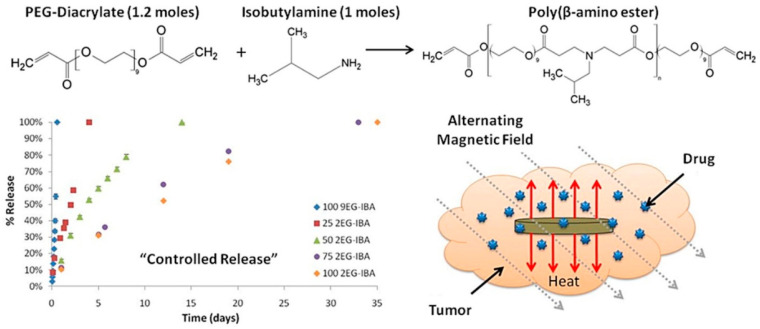
Synthesis of magnetic hydrogel for hyperthermia cancer treatment and controlled drug release [130] © 2021 Elsevier.

Hyperthermia treatment is performed at 41–46 °C alongside chemotherapy or irradiation to achieve better results in pancreatic cancer and glioblastoma [120,121,122]. However, the precise control of tissue temperature is still a difficult task for clinicians [121,123,124]. For several decades hyperthermia has been used as a radiosensitizer and chemosensitizer, resulting in significant improvements in cancer diagnosis and treatment. This combined hyperthermia technique has been demonstrated to be highly successful in the treatment of malignancies such as bladder cancer, cervical cancer, breast cancer, head–neck cancer, melanoma, and soft-tissue cancers. The mechanism of hyperthermia can be explained as the delivery of heat to the affected region, but it can be performed in various ways. Hyperthermia directly affects the cellular components and delays lethal activity towards cellular responses. DNA repair pathways and a good systemic immune response are among the activities observed in cells following heat treatment. Furthermore, heat affects hypoxic and nutrient-depleted tumor regions, whereas radiation and chemotherapy do not require such monitoring. Besides these, hyperthermia also affects tumor growth, oxygen supply pathways, and vascularization. However, there are three important considerations to keep in mind when using hyperthermia in clinical systems, as indicated by clinicians: First, the temperature rise should be exact and focused. The second step is to regulate the temperature in the affected area, rather than in other parts of the tumor. The last one is the optimization of heat/dose of the hyperthermia, as per the condition of the patient’s body. Hyperthermia systems are quite wide depending on the applied frequency ranges: they are classified as radiofrequency (RF), ultrasound, infrared (IR), and microwave (>300 MHz). The formation of eddy currents (for high electrically conducting samples), magnetization reversal (for magnetic materials), and dipolar motions of magnetic dipoles might all be part of the hyperthermia mechanism. Eddy current production is an outcome of low induction and is not restricted to magnetic materials. It is often used for a wide range of macroscopic materials with high electrical conductivity. When an electrically conducting material is subjected to an alternating material field, an eddy current is created (AMF). A Brownian connection is used for magnetic dipolar movement, resulting in heat generation. The system for MGHs, on the other hand, is a composite in which MNPs are detained but the polymer chains are not. Polymer macrochains are physisorped on the surfaces of MNPs, followed by the full restriction of rotation and movement. As a result, the Brownian relaxation process is not one of the established mechanistic paths proposed by researchers. Néel relaxation is the adopted hypothetical way to explain the heat generation inside MGHs. MHGs provide better results in this context because of their tissue mimetic behavior and remote control of intrinsic features [125,126]. As previously reported, a PVA-based magnetite-MNP-loaded composite hydrogel demonstrated a rapid temperature rise [127]. When a 357 kHz alternating magnetic field was applied, the temperature rose from 43 °C to 47 °C in 5–6 min, according to this study. From the result it was also inferred that the heating efficiency was directly related to the MNPs present in the system. Similarly, in another work Fe_3_O_4_ microparticles were used to prepare PNIPAM-based thermoresponsive hydrogels [128]. The specific adsorption rate (SAR) is an important measure for hyperthermia researchers. The quantity of heat emitted by a substance in a given amount of time is known as the SAR. It is also dependent on the external magnetic field strength. It is mathematically defined as c(ΔT/Δt), where ‘c’ and ‘ΔT/Δt’ correspond to the specific heat capacity and time-dependent temperature increment, respectively. It is critical to increase or improve the SAR value by as much as is feasible. The SAR is affected by a number of elements, including the intensity of the external magnetic field, the frequency of the alternating current, the permeability of the particles under test (in this case, MNPs), and the shape and size distribution of the MNPs. Anderson et al. fabricated PEG-based MHGs which showed temperature rising at the hyperthermia range as well as in the thermoablation range (61–64 °C) [129]. In their work they showed that cell necrosis was observed against gliobastoma cells. The same group also reported a poly(β-amino ester)-based biodegradable hydrogel (Figure 7) for hyperthermia treatment where the hydrogel was remotely controlled by an external magnetic field [130].

**Figure 8:**
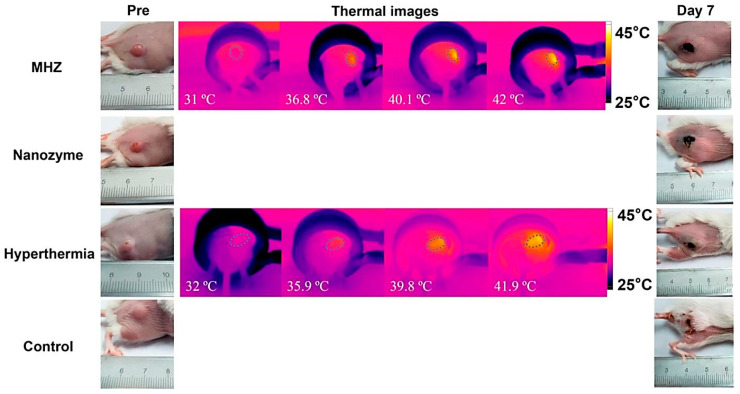
Tumor treatment process by hyperthermia. Here, the magnetic nanoenzyme was utilized as the target-specific material which was stimulated in the presence of an alternating magnetic field [131] © 2021 American Chemical Society.

Besides hyperthermia-based drug delivery, MNP-based hydrogels are also used as a targeted tumor treatment. The tumor microenvironment has a critical microstructure with uncommon biological features, such as acidosis and high glutathione content, compared to normal cells. Wu et al. reported a magnetic, injectable hydrogel for tumor treatment by hyperthermia [131]. They used PEGylated MNPs and cyclodextrin to prepare a nanoenzyme hydrogel which showed temperature increments of up to 42 °C. Injectable hydrogels are superior compared to traditional macroscopic hydrogels due to target-specific activity and easy reach to the infected area. Combinational therapy was also reported in this work, showing synergy between drug release and hyperthermia. They showed that the synergy of drug release and hyperthermia cured a tumor within 7 days of treatment in several intervals. The treatment was monitored by an infrared camera to evaluate the exact position of heating, as shown in Figure 8.

**Figure 9:**
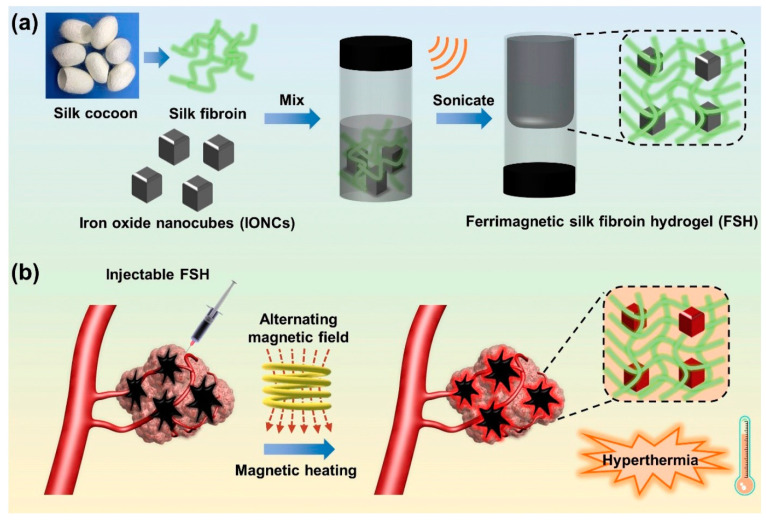
(**a**) Fabrication of a ferrimagnetic silk fibroin hydrogel by the physical attachment of PEG-stabilized iron oxide nanocubes and silk fibrion. (**b**) Prepared injectable hydrogel for liver-targeted thermoablation [134] © 2021 Elsevier.

Chen et al., fabricated a ferumoxytol-medical-chitosan-based hydrogel which showed tumor apoptosis in the presence of an alternating magnetic field [132]. Furthermore, they demonstrated that when an anti-cancer agent (in this case, doxorubicin) is coupled to the MNP-based hydrogel it improves xenograft tumor treatment effectiveness. Sol–gel transitions in hydrogel systems are another method for delivering molecular payloads and implantations to specified areas of the body without invasive paths. In this case, injectable hydrogels are appropriate since they gel quickly at body temperature. Injectable hydrogels are beneficial in the treatment of localized hyperthermia. Thermoresponsive polymers, which have been used to fabricate MHGs, are quite common in this situation. There are several limitations however, such as the adjustment and optimization of MNP concentration, viscosity of the hydrogel after the incorporation of MNPs, and undesired migration of MNPs beside the targeted site. Gelatin-based MHGs were reported for the synergistic application of hyperthermia and chemotherapy [95]. Methacrylic-anhydride-functionalized gelatin was copolymerized with 2-dimethylaminoethyl) methacrylate, with methacrylate-end-capped magnetic nanoparticles serving as the MNPs. This hydrogel has built-in magnetism and pH sensitivity, making it a dual-responsive gadget. Salloum et al. fabricated a ferrofluid-based injectable agarose gel for hyperthermia applications [133]. Qian et al. prepared a PEG-stabilized iron-oxide-nanocube-loaded silk fibroin hydrogel for antitumor therapy [134]. This hydrogel (Figure 9) showed shear thinning behavior and was applied as an injectable hydrogel. The prepared hydrogel was injected into a rabbit liver tumor and heated with an external oscillating magnetic field followed by thermoablation of the cells. Another important property of the hydrogel is its injectability, which allows for the rapid delivery of molecular payloads into specified areas via a less invasive manner. The law of sol–gel flow behavior applies to injectable hydrogels. A distinct type of hydrogel, in which substantial intermolecular interactions predominate in gel matrices, has demonstrated a solution to gelation. The physical cross-linking of these hydrogels is takes place (H-bonding, hydrophobic association, and van der Waals interactions). Jordan et al. reported injectable hydrogels based on chitosan and a block copolymer (poloxamer 407). Block copolymers show excellent sol–gel transitions with an alteration in temperature. Biopolymers and superparamagnetic iron oxide nanoparticles were employed as an additional phase in these hydrogels (SPIONs) [135]. SPIONs of 20% (*w*/*v*) were incorporated into thermoresponsive polymer matrices and injected for implantation, followed by heating with an AMF. Similarly, a 10% (*w*/*v*)-SPION-loaded biopolymer hydrogel was prepared by the ionic gelation method and injected into tumor sites. Among the block-copolymer-based injectable hydrogels, poly(ethylene-co-vinyl alcohol) (EVAL) is a significant name. An EVAL-based SPION-loaded hydrogel was reported which acted as an injectable hydrogel and showed a high SAR value when heated by an AMF [136]. Rheology is commonly used to determine injectability. SPIONs with a large surface area are prone to adsorption by polymers, limiting the flow behavior of composites. SPIONs additionally improve the thixotropic character of the material and postpone network rupturing during shear stress. When injectable hydrogels are pressed to be inserted into the body by a fine diameter nozzle, they suffer from high shear stress. The SPIONs offer the gel the strength required to hold the composite in place during the procedure without premature rupture. In SPION-based injectable hydrogels, gelation at body temperature and insolubility are also non-negotiable characteristics. In general, in specific polymer concentrations, pluronic-type hydrogels display a good transition from a solution into a gel phase. Pluronics are block copolymers that dissolve in water at room temperature. However, at a certain concentration they gel at a specified temperature, which are referred to as the critical gel concentration and critical gelling temperature. As strength is the primary quality of any injectable, filler particles are introduced. SPIONs act as a reinforcement in the hydrogel and also maintain their dimensional integrity inside the body. Moreover, the heating capability of such hydrogels is also not compromised. For injectable MHGs, the target and press ion are much more accurate than the macroscopic hydrogels of bulks. These MHGs can be injected into the exact location and easily heated by an AMF.

## 6. Applications in Drug Delivery

Hydrogels are soft materials arranged in three dimensions by hydrophilic polymeric networks [137]. Hydrogels have several unique physical features due to their porous structure, making them a good material for drug couriers and controlled release of drug molecules under specified environmental conditions [138,139]. The release of drug molecules from a hydrogel matrix is dependent on their diffusion behavior [140,141]. Drug molecules are imbibed into the hydrogel network and captured by the hydrogel matrix when polymeric hydrogels are submerged in a drug solution [142]. During some special environmental conditions the drug molecules come out from the hydrogel matrix by obeying Fick’s diffusion law or some anomalous diffusion kinetics [143]. Such behavior is also shown by different nano-drug carriers [144,145]. In the presence of some external stimuli, most hydrogels are particularly vulnerable to having their internal microstructure and porosity manipulated [146]. The most common stimuli for hydrogel systems are the pH of a solution, an electric field, temperature, a saline environment, light, enzymes, and a magnetic field [147,148]. Among the external stimuli, the magnetic field is comparatively new with respect to the others. The magnetic field might be constant or variable in frequency. A frequency-dependent magnetic field, also known as an alternating magnetic field or an oscillating magnetic field, is one in which the magnetic dipoles are activated, causing the magnetic nanoparticles to be heated.

**Figure 10:**
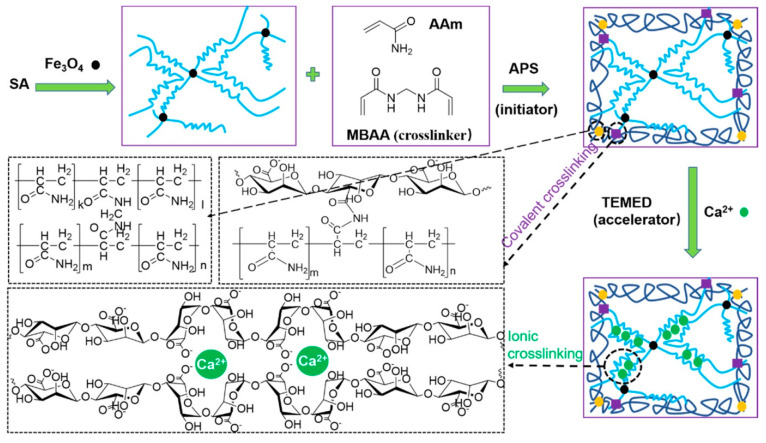
Schematic of reaction pathway to prepare a sodium alginate (SA)-based double-network magnetic hydrogel. During the gelation of ammonium persulfate (APS) *N,N,N′,N′*-tetramethylethylenediamine (TEMED) and *N,N′*-methylenebis(acrylamide) (MBAA) were used as an activator and cross-linker, respectively [154] © 2021 American Chemical Society.

Gelatin and magnetic nanoparticles were combined together to prepare a magnetic composite hydrogel where genipin was used as a cross-linking agent [149]. Vitamin B12 was utilized as a model payload in this hydrogel system, and it diffused out of the hydrogel matrix in the presence of an external magnetic field. The amount of vitamin B12 released from the hydrogel matrix was proportional to the duration of the magnetic field. This suggests that MHGs might be regulated by external magnetic fields, which could govern the pace of release and dosage. In another work, chitosan and sodium alginate were taken to fabricate MHGs regulated by an external magnetic field [150]. The author employed a responsive polymer in this ferrogel to induce temperature-dependent medication release. Another set of researchers reported carboxymethyl cellulose (CMC)- and iron-oxide-based nanocomposite MHGs [151]. In this work they also showed how external magnetic fields can influence the cumulative release percentage of any model drug. A 2-hydroxyethyl methacrylate and iron oxide composite hydrogel has previously been reported to prepare microrobots to deliver anti-cancer drugs to a specific section. Huang et al. described an MNP-based copolymer hydrogel with triple-responsive behavior to pH, temperature, and glucose [152]. These hydrogels showed self-regulatory drug release and superparamagnetic behavior. Tragacanth gum (TG)- and poly(acrylic acid)-based MHGs were reported to prepare a smart drug delivery system [153]. Here, the MNPs were magnetite and the hydrogel showed cell apoptosis against a HeLa cell line. Cao et al. developed double-network MHGs from polyacrylamide and alginate [154]. The hydrogel was tough and compressible (Figure 10). This was used in magnetic robots in underwater applications as well as an efficient drug delivery device. They proposed a straightforward method for making magnetic hydrogels with good mechanical properties by combining physical mixing and chemical cross-linking procedures in their work. MNPs were fine-tuned and a fast magnetic response was created. The magnetic hydrogel was utilized to make two standard magnetically responsive marine animal robots (a scallop and a starfish) that were used to clean the fish tank using a remotely controlled magnet. The suggested technique may be applied to different hydrogel systems, expanding the range of smart hydrogel applications.

**Figure 11:**
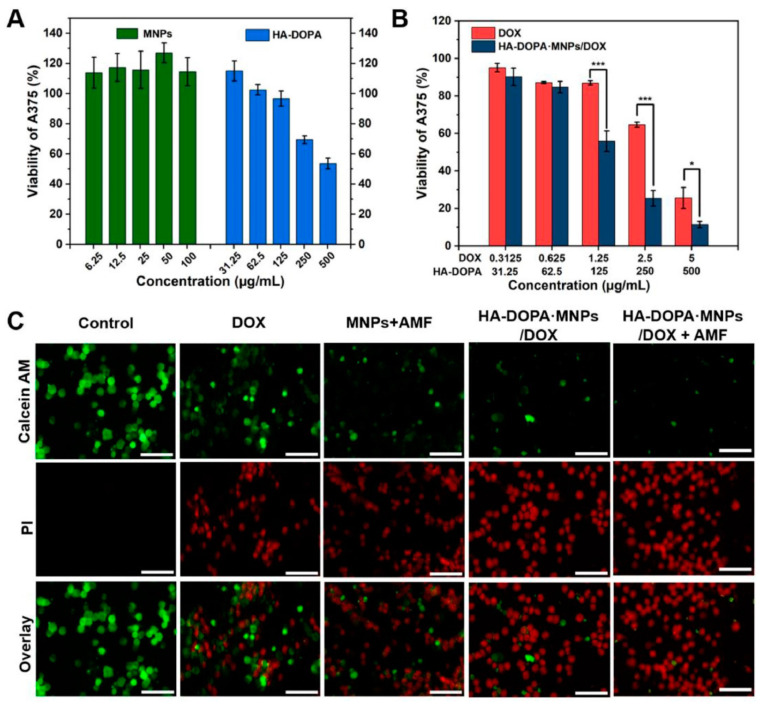
(**A**) Viabilities of human melanoma cells (A375) after 24 h of treatment with MNPs and an injectable hydrogel made of dopamine-conjugated hyaluronan (HA-DOPA). (**B**) Viabilities of A375 cells after 24 h of treatment with an anti-cancer drug (doxorubicin; DOX) and an anti-cancer-drug-loaded hydrogel. (**C**) Fluorescence images of A375 cells with staining of calcein AM (AM, green, live cells) and propidium iodide (PI, red, dead cells) after various treatments. The images show how magnetic fields and anti-cancer drugs both act on cell death. Scale bar: 100 µm [155].

In another work, a nanocomposite hydrogel was prepared from the dopamine–Fe^3+^ complex and reinforced with MNPs [155]. The authors discovered that MNPs had a significant impact on their shear modulus. They tried a combinational approach for the cancer treatment. They mingled the hyperthermia and targeted drug delivery into one system and showed better efficacy towards cancer treatment. The hydrogel was regulated by the external magnetic field and could be heated in a non-contact mode. When an external AMF was turned on, the nanocomposite hydrogel showed a pulsed release of an anti-cancer drug (DOX), but when the AMF was turned off it reverted to its slow releasing mode. In addition, in vivo, the DOX-loaded composite hydrogel had a longer retention duration than the DOX-loaded gel or DOX solution. They also hypothesized whether if a single-modal treatment was carried out, i.e., only with an anti-cancer drug or hyperthermia, the curing would be as effective as the combined synergy (Figure 11). The live–dead assay of the cell line also implied that magnetic fields and anti-cancer drugs together can cause cell death easier than using a single tool.

## 7. Outlook and Future Remarks

Stimuli-responsive hydrogels are within the category of magnetically controlled smart hydrogels. Their fabrication and two of their major applications were discussed in this article. Polymers are non-magnetic in general; however, they become magnetic when magnetic nanoparticles are added. To assess their mode of work and application, the magnetization value acquired from their saturation magnetization values is critical. In this paper, we looked at two biomedical applications in which magnetic composite hydrogels are utilized to transport tiny molecules such as drugs, vitamins, or other physiologically important payloads. Magnetic heating, often known as hyperthermia, is another use. The magnetic nanoparticles are activated by an external alternating magnetic field, which causes the nanoparticles to heat up. Because they were inside the hydrogel, the entire hydrogel served as a heat reservoir. As heat and medication delivery were coupled, their activity increased when compared to when they were used individually. Polymeric systems based on MNPs have been employed in a variety of applications.

Magnetic hydrogels benefit from their superparamagnetic and heating properties when subjected to an AMF. When adjusting the degree of deformation, reaction qualities are important. To change the swelling state and degradation rate, the aspects of an MF on the outside (e.g., intensity and frequency) must also be changed. As a result of this, rapid MNPs and polymers with higher molecular weights are the only ones that produce a reaction. Hydrogels can be made from biocompatible and biodegradable materials. As a result, magnetic hydrogels might be used to design 3D complex tissue structures using bottom-up assembly methods, fabricate soft actuators, regulate temperature, and focus cancer therapy to the tumor site.

There is a much in the way of potential for merging real-time diagnostic methods with intelligent therapeutics. Such a potent combination is especially appealing for hyperthermia-based therapy and drug administration utilizing MNPs, where imaging technologies such as MRI and fluorescence imaging may be incorporated to generate very intelligent theranostics. Future research will lead to the growth of intelligent theranostics for noninvasive drug pharmacokinetics and pharmacodynamics as well as real-time therapeutic response monitoring. We believe that biomedical nanotechnology, in general, and hyperthermia-based therapy as well as drug delivery techniques based on magnetic nanoparticles, in particular, will help the pharmaceutical industry shift away from the burst release drug prototype and toward custom-made medicine.

## Figures and Tables

**Table 1:** Various types of magnetic hydrogels and their mode of synthesis.

Hydrogel Matrix	MNPs	Concentration	Method	Ref.
Chitosan	Fe_3_O_4_	11.1–13.6 wt%	In situ	[117]
Alginate/PNIPAM	γ-Fe_2_O_3_	-	In situ	[106]
PAAm-GA	Fe_3_O_4_	8.3–14.04 wt%	In situ	[104]
Fibrin	Fe_3_O_4_	2.5 mg/mL	Blending	[60]
Dextran	CoFe_2_O_4_	2.5–15 wt%	Blending	[92]
Alginate	FePt	8 wt%	Blending	[118]
PNIPAM	CoPt	1 wt%	Blending	[91]
PAAm	CoFe_2_O_4_	2 wt%	Grafting onto	[114]
NIPAM	Fe_3_O_4_	<50%	Grafting onto	[119]
NIPAM	γ-Fe_2_O_3_	~50%	Grafting onto	[119]
CMC	CoFe_2_O_4_	1.5 wt%	Grafting onto	[113]

PAAm—polyacrylamide; PNIPAM—poly(N-isopropylacrylamide); GA—gum acacia; NIPAM—N-isopropylacrylamide; and CMC—carboxymethyl cellulose.

## Data Availability

Not applicable.
